# Antibiotic Stewardship at transitions of care: identifying opportunities for antibiotic optimization

**DOI:** 10.1017/ash.2026.10305

**Published:** 2026-03-25

**Authors:** Corey Johnson, Jenni Thomas, Sara Valanejad, Corey Medler

**Affiliations:** 1 Cone Health Moses Cone Hospital, Greensboro, USA; 2 University of Pittsburgh Medical Center East, Pittsburgh, USA; 3 University of Virginia Health System, Charlottesville, USA

## Abstract

We conducted a cross-sectional study to identify the frequency of optimized oral antibiotic regimens at transitions of care (TOC) in patients diagnosed with respiratory, urinary tract, or skin structure infections. An optimal regimen was identified in 62.5% of patients at TOC, highlighting opportunities for antimicrobial stewardship interventions at TOC.

## Background

Antimicrobial stewardship programs (ASP) have become a focal point in the hospital setting to reduce unnecessary antibiotic use amongst the growing concern over antibiotic resistance.^1^ An estimated 50% of hospitalized patients receive antibiotics during their admission with roughly 20% of all patients prescribed antibiotics at discharge.^2^ Hospital-based ASP interventions have been shown to improve inpatient prescribing practices by utilizing strategies such as prospective audit and feedback and preauthorization. However, these practices may not translate into prescribing at time of hospital discharge.

Prior studies have demonstrated that a significant portion of antibiotic prescriptions written at hospital discharge are inappropriate, often due to excessive durations of therapy.^3–5^ The transition of care (TOC) from the hospital setting to the outpatient setting represents a key opportunity for antibiotic therapy reassessment. This study aims to identify the frequency of optimized antibiotic regimens at TOC and assess the potential for implementing a quality improvement project to improve antibiotic prescribing on discharge.

## Methods

This was a retrospective, cross-sectional, study evaluating oral antibiotic appropriateness at hospital discharge from an academic hospital between July 1, 2021 and June 30, 2023. Patients were included if diagnosed with a respiratory tract infection (RTI), urinary tract infection (UTI), or skin and skin structure infection (SSSI) and discharged on oral antibiotics from a general medicine service. Patients ≤18 years old, immunocompromised patients (ANC <500 or CD4 count <200), solid organ transplant patients, and patients discharged on intravenous (IV) antibiotics were excluded.

The primary endpoint was the frequency of optimized regimens in patients discharged with oral antibiotics for the treatment of uncomplicated RTI, UTI, or SSSI. RTIs included community-acquired pneumonia, hospital-acquired pneumonia, or chronic obstructive pulmonary disease exacerbation. UTIs included cystitis, complicated UTIs, or pyelonephritis. SSSIs included non-purulent drainage/discharge, purulent drainage/discharge, or abscess with source control.

Institutional and national guidelines (i.e., Infectious Diseases Society of America [IDSA]) and evidence-based best practices were used to assess the appropriateness of antibiotic selection, dose, and duration. Patient regimens were categorized as optimal or non-optimal. Non-optimal regimens were subcategorized as unnecessary, inappropriate, or suboptimal based on definitions proposed by Spivak, et al.^6^ An optimal regimen was defined as use of antibiotics for an established bacterial infection and containing an appropriate agent, dose, and duration. An unnecessary regimen included antibiotics for nonbacterial infections, redundant antibiotic treatment, or duration therapy beyond indicated use without clinical rationale. An inappropriate regimen was defined as use of specific antibiotics for which the pathogen was resistant, the use of antibiotics was not recommended in treatment guidelines, or the duration of therapy was less than clinically indicated. Regimens in the setting of an established bacterial infection that could be improved through drug choice, route, or dose were considered suboptimal.

### Data collection

Patients identified through the institution’s electronic medical record (Epic Systems Corporation) and randomized. Data were manually collected using a standardized REDCap case report form, including demographics, antimicrobial agent, and prescribing characteristics. Data were collected by a single author and reviewed by additional authors.

### Statistical analysis

All statistical analyses were performed using SPSS Software, version 28.0 (SPSS, Inc). Descriptive measures included incidence, proportions, and measures of central tendency and dispersion.

## Results

There were 659 patients screened with 200 patients that met inclusion criteria. The primary reason for exclusion was antibiotic indication outside the scope of this project. Patient characteristics are summarized in Table 1. The indication for antibiotics prescribed at discharge was UTI in 79 (39.5%) patients, RTI in 66 (33%) patients, and SSSI in 55 (27.5%) patients (Table 1).

**Table 1.** Patient characteristics and antibiotic indications

Characteristics	Patients (*n* = 200)
**Patient characteristics**	
Gender (female)	110 (55%)
Age (y) (median, IQR)	67.5 (55–79)
Charlson comorbidity index (median, IQR)	1 (0–3)
**Antibiotic indication**	
**UTI**	**79 (39.5%)**
cUTI	37 (18.5%)
Cystitis	32 (16.0%)
Pyelonephritis	10 (5.0%)
**RTI**	**66 (33.0%)**
CAP	53 (26.5%)
COPD Exacerbation	10 (5.0%)
HAP	3 (1.5%)
**SSSI**	**55 (27.5%)**
Non-purulent	36 (18.0%)
Purulent	10 (5.0%)
Abscess w/ source control	9 (4.5%)

Note. UTI, urinary tract infection; RTI, respiratory tract infection; SSSI, skin and skin structure infection; cUTI, complicated urinary tract infection; CAP, community-acquired pneumonia; COPD, chronic obstructive pulmonary disease; HAP, hospital-acquired pneumonia.

An optimized antibiotic regimen at TOC was identified in 62.5% of regimens (*n* = 125). This resulted in 37.5% of regimens that were identified to have at least one opportunity to improve antibiotic selection, dose, and/or duration. Antibiotic regimens were categorized as unnecessary in 52 (26%) patients, suboptimal in 33 (16.5%) patients, and inappropriate in 7 (3.5%) patients. For patients categorized as receiving unnecessary therapy, 51 (98.1%) patients received an antibiotic duration of therapy extending beyond clinical indication (Table 2).

**Table 2.** Categorization of optimal vs non-optimal therapy and evaluation of antibiotic duration

	All (*n* = 200)	UTI (*n* = 79)	RTI (*n* = 66)	SSSI (*n* = 55)
**Categorization of regimen**				
**Optimal therapy**	**125 (62.5%)**	**49 (62.0%)**	**43 (65.2%)**	**33 (60.0%)**
** Unnecessary therapy**	**52 (26%)**	**21 (26.6%)**	**12 (18.2%)**	**19 (34.5%)**
DOT beyond clinical indication	51 (98%)	20 (25.3%)	12 (18.2%)	19 (34.5%)
Miscounted days	23 (45.1%)	6 (7.6%)	7 (10.6%)	10 (18.2%)
No documented rationale	17 (33.3%)	8 (10.1%)	3 (4.5%)	6 (10.9%)
New antibiotic course started at discharge	11 (21.6%)	6 (7.6%)	2 (2.5%)	3 (5.5%)
Total unnecessary days	208 days	85 days	34 days	89 days
**Suboptimal therapy**	**33 (16.5%)**	**16 (20.3%)**	**13 (19.7%)**	**4 (7.3%)**
Therapy too broad following culture result	15 (45.5%)	9 (56.3%)	2 (15.4%)	4 (100%)
Delay switch to PO antibiotics	14 (42.4%)	4 (25.0%)	11 (84.6%)	0 (0%)
Incorrect dosing regimen	3 (9.1%)	3 (18.8%)	0 (0%)	0 (0%)
Total suboptimal days	136 days	74 days	27 days	35 days
**Inappropriate therapy**	**7 (3.5%)**	**3 (3.8%)**	**3 (4.5%)**	**1 (1.8%)**
Use not recommended in treatment guidelines	4 (57.1%)	3 (100%)	1 (33.3%)	0 (0%)
DOT less than clinical indication	3 (42.9%)	0 (0%)	2 (66.7%)	1 (100%)
Total inappropriate days	33 days	20 days	9 days	4 days
**Median duration of antibiotic therapy by indication, days (IQR)**				
Total duration	N/A	7 (6–9)	5 (5–7)	9 (7–11)
Unnecessary days	3 (2–5)	3 (2–6)	2 (2–4)	4 (2–7)
Inappropriate days	5 (2–7)	7 (5–7)	2 (2–2)	4 (4–4)
Suboptimal days	3 (2–6)	4 (2–7)	2 (1–3)	8 (8–11)

Note. UTI, urinary tract infection; RTI, respiratory tract infection; SSSI, skin and skin structure infection; DOT, duration of therapy; PO, oral.

In cases where antibiotic duration extended beyond clinical indication, the primary reason was due to a miscount of antibiotic days (*n* = 23; 45.1%). Antibiotic durations were discordant with institutional/national guidelines in 17 (33.3%) patients and 11 (21.6%) patients had their antibiotic course restarted at discharge after receiving active therapy while hospitalized. In patients categorized as receiving suboptimal therapy, 15 (45.5%) patients had broader therapy than indicated following one day of culture results and 14 (42.4%) patients had an opportunity to be transitioned from IV to an oral regimen based on clinical improvement and ability to take medications by mouth. For patients categorized as receiving inappropriate therapy, 4 (57.1%) patients received therapy that did not align with institutional/national guidelines and 3 (42.9%) patients received an insufficient antibiotic duration.

Overall, patients had a median (IQR) of 3 (2–5) days of unnecessary therapy, 5 (2–7) days of inappropriate therapy, and 3 (2–6) days of suboptimal therapy (Table 2). Excessive duration was identified in 25.5% of patients at discharge. Regimens associated with SSSIs were identified as having the greatest number of excessive days with a median (IQR) of 4 (2–7) days longer than guideline recommendations. This was followed by UTI with a median (IQR) of 3 (2–6) excessive days with pyelonephritis having the highest median (IQR) of 6 (5–9) excessive days. Lastly, regimens prescribed for RTI resulted in a median (IQR) of 2 (2–4) days of excess therapy beyond clinical indication (Table 2).

## Discussion

This study aimed to evaluate antibiotic prescribing practices at TOC, specifically focusing on identifying stewardship opportunities at discharge. Our findings underscored the prevalence of non-optimal antibiotic regimens prescribed on discharge. We found that 62.5% of antibiotic regimens were classified as optimized, leaving 37.5% of regimens with opportunities for improvement. The primary reason for regimens being deemed unnecessary was the extension of therapy beyond clinical indication, accounting for 25.5% of regimens.

Limitations of this study included information bias as results were reliant on accuracy and completeness of information provided in patient charts.

Antimicrobial stewardship (AS) at TOC can be implemented through a variety of interventions based on hospital specific resources. Key elements include confirming the infectious diagnosis still applies at discharge, using the shortest effective duration of therapy, using the narrowest effective spectrum of activity, and improved communication.^7^ For resource limited hospitals, targeting specific diseases states or antibiotics associated with higher toxicity rates may increase likelihood of a successful intervention.^8^ Mercuro et al implemented a pharmacist-led intervention where pharmacists collaborated with physicians to optimize oral antibiotics regimens at discharge for UTI, RTI, SSSI.^9^ Patients were more likely to have an optimal antibiotic prescription in the postintervention group and had no difference in clinical resolution or mortality.^9^

This study revealed substantial gaps in antibiotic prescribing practices at TOC, with a significant number of regimens being unnecessarily prolonged or inappropriate. Institutions can reduce unnecessary antibiotic exposure by implementing targeted interventions, education, and/or evidenced-based guidelines. ASP interventions at discharge demonstrate significant benefits, including use of more narrow spectrum agents, shorter durations of therapy, and fewer adverse drug events. Evaluating antibiotic prescribing practices at discharge offers a pivotal opportunity to assess gaps in care and ascertain AS opportunities.
